# Supplementary figures and images for: Resting-state brain metabolic fingerprinting clusters (biomarkers) and predictive models for major depression in multiple myeloma patients
Source: PLoS One. 2021 May 6;16(5):e0251026. doi: 10.1371/journal.pone.0251026 (PMC8101966; doi:10.1371/journal.pone.0251026)

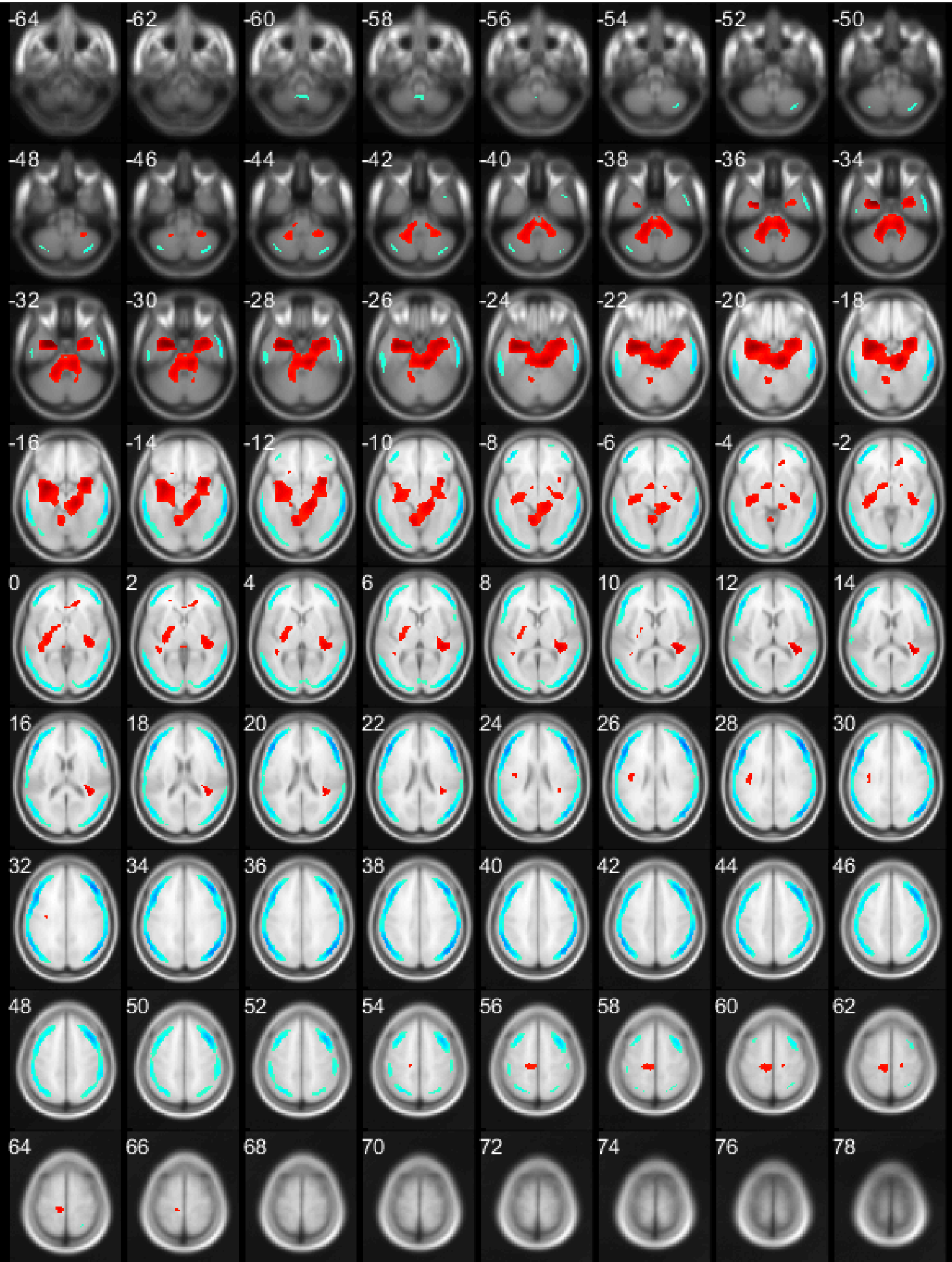

Supplement: S1 Fig — Statistical parametric maps displayed on transverse sections depicting regions of hyper-metabolic (red) and hypo-metabolic (blue) changes. (TIF) [file pone.0251026.s001.tif]
