# Supplementary material for: Resting-state brain metabolic fingerprinting clusters (biomarkers) and predictive models for major depression in multiple myeloma patients
Source: PLoS One. 2021 May 6;16(5):e0251026. doi: 10.1371/journal.pone.0251026 (PMC8101966; doi:10.1371/journal.pone.0251026)
Supplement: Attachment — Submitted filename: Response to Reviewers.docx [file pone.0251026.s002.docx]

Matteo Bauckneht, M.D., Ph.D

Academic Editor
PLOS ONE

[Submission Date: Month Day, Year]

Dear Dr. Bauckneht:

Thank you for inviting us to submit a revised draft of our manuscript entitled, "Resting-state brain metabolic fingerprinting clusters (biomarkers) and predictive models for major depression in multiple myeloma patients " to PLOS ONE. We also appreciate the time and effort you and each of the reviewers have dedicated to providing insightful feedback on ways to strengthen our paper. Thus, it is with great pleasure that we resubmit our article for further consideration. We have incorporated changes that reflect the detailed suggestions you have graciously provided. We also hope that our edits and the responses provided below satisfactorily address the issues and concerns you and the reviewers have noted.

To facilitate your review of our revisions, the following is a point-by-point response to the questions and comments delivered in your letter dated March 10, 2021.

Reviewer #1: The manuscript faces an interesting, though not commonly observed, topic. Here below there are some suggestions and some corrections that should be made, in my opinion, in order to make it eligible for publication.

1. Errors: Thank you for the suggestions. We have corrected the errors below (a-g). We have clarified that the line 235 (g) states the MNI coordinates of clusters with adding (x, y, z) (ln 252).
a) Line 57: please substitute "(Satin 2009)" with the progressive reference number and then modify the list of refs.;
b) Line 62: as above, please replace the link with the reference number;
c) Line 74: please correct "have developed" with "have BEEN developed";
d) Line 78: please repeat the acronym "PET/CT" in place of the full name;
e) Line 86: did you mean "approximately 24% of multiple myeloma PATIENTS suffered from"?
f) Line 161: please replace the link with the reference number;
g) Line 235: please put all the reference numbers together.

2. Aspects that should be better clarified:
a) M&M section: the modality of acquisition of brain PET images must be explained. For example, was it a separate bed acquisition? What was the duration of the brain scan? Did you use any immobilization system to avoid motion artifacts? How did you orient patient's head? We have expanded M&M section in detail. In brief, we used the the PACS’s stack tool to extract the brain FDG activity from the standard whole-body FDG PET/CT without immobilization. Using SPM, we then reorient the subject’s brain images to the MNI template one by one.

b) Results Section: please describe more in detail the areas that have shown to differ from group 1 and 2; Thank you for your suggestion. You may have noticed that the sizes of clusters was determined by the cutoff T values of the T-contrast maps as compared in Fig.3 and S1 Fig. We did not emphasize the area in the result section but described the clusters more in discussion section when we compared our results to others.
c) Line 224: please explain the link between glutamate neurotransmission, FDG uptake and major depression; This is an interesting perspective. We have added the following sentence to Discussion: “It has been estimated that as much as 70 % of the energy derived from glucose oxidation is required to convert glutamate, the major neurotransmitter in the brain, to glutamine during glutamatergic transmission. Therefore, the FDG PET signal is proxy for measuring excitatory neurotransmission”.
d) Please add a paragraph/a few sentences about the limits of your study. Thank you for the suggestion. We have added a Limitations sections to the manuscript (p 15).

Reviewer #2: Thank you very much for an interesting paper regarding the use of FDG PET for predicting major depression in cancer patients. I appreciate the retrospective use of PET data for assessing additional clinical use of the functional metabolic data.

I will not address the postprocessing of the scans, since this is not my field of expertise. I will rather comment on the differences between the two groups.

3. On a general note to the changes observed in brain glucose metabolism in the depression group: could the findings also/additionally be explained by possible patient anxiety or maybe more likely by ongoing medication/chemotherapy? You have raised an important question. We have incorporated this suggestion throughout our paper, and highlighted this in our Limitations paragraph.

4. And for that matter, are data for patient diabetes, BS levels/fasting compliance available? The insulin levels are very important for global and regional uptake of FDG. Also where the patients blindfolded/in a quiet room during the uptake period? Thank you for the fair questions. We have included the number of diabetic patients and patient’s BS levels at the time of injection in Table 1. Patients were injected in a quiet, dimly lit, warm room; however, they were not blindfolded. We have added this information to Methods section.

5. Are there known differences in morbidity between the groups except the major depression?
F.ex other mental illnesses that could mimic depressive metabolic reductions in the cortex of the brain. While there were no known additional differences between the two groups, this is an important consideration and has been added to the Limitations section.

6. I would prefer a few comments considering these above mentioned circumstances, preferably addressing them as possible biases. Please see point 5 above.

7. Regarding the conclusions. I would prefer a few lines addressing the possible use of the conclusions achieved. Could you imagine all brain PET data from cancer patients going through a screening for major depression using your predictive model? Yes, the eventual progression of these methods is to screen all patients undergoing PE/CT for major depression. With the rapid advancement of AI, there is potential for automated screening of patients during the staging PET/CT for major depression.

8. Would it not be more feasible to recommend a psychological evaluation of all patients and the PET data as an add on? This would not replace the need for a psychological evaluation; however, this may alert the clinician to metabolic changes in the brain that have been associated with depression. The patient may have sub-clinical depression or may have been embarrassed to express their symptoms to their oncologist. This would serve as a supplemental screening exam to the more traditional questionnaires.

9. And a side note: In our PET department the brain is not scanned in cancer patients on a regular basis to spare radiation to the lens of the eyes. In your experience, how many FDG PET scans in cancer patients include the brain as well? This is a valid concern. We do a disproportionate amount of whole body PET/CT’s because of our large multiple myeloma population. We have approximately 60 whole-body FDG PET/CT scans (50% of our week workload) for Multiple Myeloma per week.
10. More specific comments:

a) P. 5, l. 100: Were the 96 patients screened for depression? Patients were not screened for depressive symptoms at the time of the exam and is a limitation of the study. See point 5.
b) P. 12, l. 220: I would like this statement explained. Did the cancer patients with major depression worsen? And did patients without major depression develop it afterwards? Thank you for the important question. We noted that the major depression group had less global FDG activity than the control group. By searching Pubmed, we found Kimbrell’s report (new ref. 35), which compared 11 severely depressed unipolar patients to 11 healthy subjects with Cohen’s d (-0.83). Our two group’s Cohen’s d is -1.33; logically suggesting the patients in our study might have severe depression. Since this is a single institute, retrospective study with limitations and potential biases, further studies are needed to assess the relationship between the global/regional FDG uptake and severity of depressive symptoms.

c) P.12, l. 222: This is a repeated statement from p.11. The repeated statement has been deleted.

d) P.13, l. 243 In ref 11, the limbic lobe was a hypo-metabolic area in major depression patients. In your data it is a hyper metabolic cluster, what could be the reason for this? This is a very good question. Specifically, the study by Kimbrell et al. (cited in ref 11) which demonstrated hypometabolic changes in the limbic lobe, differing from our findings and others, was done in patients performing an auditory continuous performance task (CPT) for 30 min during the FDG uptake period. Another study by van Heeringen et al. (ref 44) showed that patients with major depression without anxiety have significantly increased FDG uptake in the left parahippocampal gyrus. Further studies are needed to assess the effect of confounding variables, such as coexisting anxiety disorders and treatment effects, on FDG uptake within this region.

Reviewer #3: The paper in general terms is understandable but it is recommended to have some additional edition because there are some points in the methodology and results that were not completely clear ; the methodology, as mentioned, is not completely clear and the “discussion” the seems very general to me and not well elaborated (Authors focus on the explanation of their results rather than providing a bunch of possible explanations. Why do the authors choose this machine learning algorithms ? ).

10. Materials and Methods
Subjects:
a) The authors mentioned ‘FDG PET/CT images from 138 patients met the criteria. Thirty-eight patients with major depression as group 1 (D), and 96 patients without major depression as group 2 (Control)’. It is not very clear what are diagnostic criteria for major depression group and without major depression group? Thank you for your questions. We have edited Materials and Methods section to address these questions. In brief, ICD 10 codes of multiple myeloma and major depression in patients’ age 30 to 65 years old were used as selection criteria.
b) It is unreasonable that you draw a conclusion only with the two groups. A healthy control group should be added. You have raised an important point. In the present study, a healthy control group is not feasible as we do not have a cohort of healthy patients undergoing whole-body PET/CT. We therefore chose patients with multiple myeloma but without the diagnosis of major depression as the control. We have added this to Limitations.
PET images:
c) The data are from different PET/CT system ,and reconstructed using different method. It is known that PET data are affected by these differences, esecially for voxel-wised analysis. it would be taken in consideration in the SPM analysis. We agree with you that different imaging reconstruct algorithms affect the SUV values, for example, GE VPHDS vs QCHD. To compare images from different systems, we used SPM proportional scaling intensity normalization to minimize the differences. We have edited this to M&M section.

11. Discussion:
Though the potential interest in Major depression has been previously reported, the authors do not clearly state what the potential relevance of Major depression among multiple myeloma patients. This should be stated more clearly so that the motivation of the study is readily apparent to the reader. Thank you for your suggestion. We want to explore the feasibility of evaluating cancer patients for major depression using whole-body oncology FDG PET/CT. Multiple myeloma is one of the cancers which requires whole-body FDG PET/T to detect bone lesion from vertex to toes. To this end, this represents an excellent data source for data mining and potential AI translational research in screening for depression in cancer patients. We have added this to our Conclusion, and we hope you will agree with us.

**CONCLUDING REMARKS**: Again, thank you for giving us the opportunity to strengthen our manuscript with your valuable comments and suggestions. We have worked hard to incorporate your feedback and hope that these revisions satisfy the concerns raised.

Sincerely,

Xiaofei Wang, MD & PhD

Corresponding Author

Chief, Nuclear Medicine Molecular Imaging and Target Therapy

Director, Nuclear Medicine Residency Program

Director, The Quantitative Lab of Functional Molecular Imaging Radiology, UAMS

Central Building, Rm 1/216B

4301 W. Markham Street – Slot 556

Little Rock, AR 72205-7199

Tel:501-686-8233

Email: xwang@uams.edu
